# The influence of community cohesion on depression: the mediating role of social support and psychological resilience

**DOI:** 10.3389/fpsyt.2025.1638958

**Published:** 2026-01-14

**Authors:** Pei Fuhua, Wang Yuanyuan, Qi Shisan, Wang Junxiu

**Affiliations:** 1 School of Psychology, Inner Mongolia Normal University, Hohhot, China; 2 School of Educational Sciences, Xinjiang Hetian College, Hetian, China; 3 Qufu Normal University, Qufu, China; 4 School of Mental Health, Wenzhou Medical University, Wenzhou, China

**Keywords:** community cohesion, depression, mental health, psychological resilience, social support

## Abstract

**Introduction:**

Depression brings profound suffering to individuals, families and society. Although some research has been conducted on the relationship between community cohesion and depression, there is no more research to reveal the internal mechanism by which community cohesion affects depression comprehensively. This study aims to examine the association between community cohesion and depression and explore the mediating roles of social support and psychological resilience in this relationship during a specific period (the COVID-19 pandemic) and in a specific setting (urban communities).

**Methods:**

This cross-sectional study included valid 1010 adults from the Chinese Social Mentality Survey in 5 places with a gender distribution of 41.2% male and 58.8% female. Community Cohesion Scale, the Patient Health Questionnaire-9(PHQ-9), Social Support Questionnaire and Connor-Davidson Resilience Scale were used to assess variables. Data analysis involved descriptive statistics, Pearson correlation, and mediation analysis using the SPSS macro Process.

**Results and discussion:**

Community cohesion had negative effect on depression. Both social support and psychological resilience were found to serve as independent and sequential mediators in the relationship between community cohesion and depression. This research contributes to a deeper understanding of how community cohesion affects depression, offering pathways that could be targeted in future interventions.

## Introduction

The epidemiological estimates of lifetime prevalence suggest that 33-50% of the people with a lifetime history of depression experience a depressive episode in a year (1). It has been estimated that the lifetime prevalence of depressive disorder in Chinese adults has reached 6.9%, 53% of which are patients aged over 50 (2). Depression brings profound suffering to individuals and families, impairs social functioning and economic productivity. Depression can lead to an escalated risk of disability, cognitive decline, and increased utilization of medical services among old adults (3). There is now compelling evidence that the risk of developing any mental health condition is inextricably linked to social structural conditions such as socioeconomic status, social support, as well as the neighborhood social and physical conditions in which people live (4). However, empirical research on depression in Chinese academic circles still predominantly focuses on the pathological level, with insufficient attention paid to the social attributes of depression (5). According to Bronfenbrenner’s Ecological Systems Theory, Community cohesion, as one of the community characteristics, exerts an influence on individuals’ mental state and behavior. Therefore, it’s necessary to examine the influence process of community cohesion on residents’ depression in order to provide theoretical guidance for the prevention and intervention of depression from the perspective of community.

Community cohesion refers to residential togetherness among people who share given geographical space,which can be shown in trust, shared values,and a willingness to take collective action among community members (6). Prior research has shown that a negative correlation between community cohesion and depression was found also in various contexts including among adolescents and young adults (7, 8), Chinese and European older adults (9, 10). During the COVID-19 pandemic, Neighborhood cohesion may be leveraged to mitigate pandemic impacts on depressive symptoms (11). Compared with adolescents and older adults, adults have been paid less attention on the relationship between community cohesion and depression. Besides, adults’ depression may lead to worse effects on family and society. Based on prior research, Hypothesis 1 is proposed: Community cohesion is negatively associated with adult depression.

However, the intrinsic mechanism through which community cohesion influences depression remains unclear. This study investigates the mechanisms through social support and psychological resilience. Social support most commonly refers to functions performed for a distressed individual by significant others such as family members, friends, co-workers, relatives, and neighbors (12). Community cohesion may achieve protective effects by enhancing access to instrumental help from neighbors (e.g., childcare, loaning possessions, home maintenance), as well as fostering connection and emotional support (8, 13). According to stress-buffering theory, social support may reduce the negative emotional reaction to a stressful event (12, 14). Researchers often find a significant and negative association between social support and depression, and social support appears to be protective against depression in youth and during pregnancy (15–17). Moreover, the strength of a community’s social networks and the extent to which it operates cohesively may affect the well-being of individual community members (18, 19).

Another key variable is psychological resilience which can explain the relation between community cohesion and depression. Psychological resilience was defined as a stress coping ability that enables one to recover and grow from adversity (20). Prior research has shown a positive correlation between social cohesion and resilience among survivors of natural disasters (21, 22), school children (23), people with HIV (24). Through providing meaningful contact with others and increasing the sense of purpose, community cohesion facilitates interaction and communication, which contribute to increase resilience at an individual and community levels (21, 25). Furthermore, relevant studies have demonstrated that psychological resilience can not only promote individual life satisfaction, but also counteract and buffer the negative effects of a series of adverse situations in the future and effectively reduce the risk of depression (26, 27). That’s to say, Individuals with low psychological resilience are more likely to be overwhelmed by disasters and develop depressive emotions, whereas those with high psychological resilience tend to protect themselves from experiencing symptoms of depression, enabling them to successfully navigate through adversity (28). Therefore, this study proposes that community cohesion affects adult depression through the mediating roles of social support (Hypothesis 2) and psychological resilience (Hypothesis 3) respectively.

Social support could potentially influence depression, with psychological resilience serving as a mediator. According to Conservation of Resources Theory (29), Social support functions as a critical resource that helps individuals accumulate psychological capital (including optimism, hope, esteem, etc.). Evidence suggests that social support enhances psychological resilience, thereby reducing vulnerability to emotional and behavioral disorder (30). Community cohesion strengthens social bonds among individuals, increases opportunities to establish networks and receive social support, which can offer the acquisition of coping skills to manage stress, fear, and avoidance behaviors (22). Recent research has found that social support and psychological resilience play a chain-mediating role in the relationship between empathy and depression among older adults (31). Accordingly, hypothesis 5 is proposed that Community cohesion may indirectly reduce depression levels in adults through the chain-mediating roles of social support and psychological resilience.

So far, although some research has been conducted on the relationship between community cohesion and depression, there is almost no research that explains their relationship by taking social support and psychological resilience as co-mediators. Considering the limitations of existing research, this study was conducted during a specific period (the COVID-19 pandemic) and in a specific setting (urban 100 communities), as we hypothesized that community cohesion and psychological resilience would function more prominently on depression during the pandemic, with residents exhibiting more support and the crisis serving as a litmus test for resilience. Additionally, unlike Western communities, Chinese communities are not only residential spaces but also fundamental units of urban governance.

In summary, based on the perspectives of Bronfenbrenner’s Ecological Systems Theory, Stress Buffering Theory, and Conservation of Resources Theory, this study intends to explore the influence of community cohesion on adults’ depression, as well as the mediating roles of social support and psychological resilience under the context of Chinese community and the pandemic.

## Materials and methods

### Data source and sampling procedure

The data for this study were obtained from a nationwide serial survey—the Chinese Social Mentality Survey (CSMS)—conducted by the Chinese Academy of Social Sciences (CASS). The survey was administered through the “Questionnaire Treasure” APP in January, 2021. Duringthe questionnaire collection process, screening questions, trap questions, and logic check questions were set up, and only participants who passed all of them were finally included in the sample. A total of 1,200 questionnaires were distributed in the study, and 1,010 valid samples were obtained, resulting in an effective response rate of 84.17%. Respondents were drawn from 5 places including Xi’an city in Shanxi Province, Jinnan District in Tianjin City, Zhengzhou City, Anyang City, Xuchang City in Henan Province. Detailed demographic information of the participants is provided in **Table 1**.

**Table 1:** Participant's demographic profile.

Particulars	Description	Percentage(%)/Mean
Gender	Male	41.2
	Female	58.8
Age		30.66
Education	Primary school and below	0.3
	Junior high school	0.9
	Senioer high school(including technical secondary school, vocational high school and technical school)	5.5
	Junior college(including current student)	11.2
	Bachelor's degree(including current student)	40.6
	Postgraduate (including current student)	41.5
Personal income	3000 yuan and below	44.1
	3000-7000 yuan	23.8
	7000-15000 yuan	27.4
	Over 15000 yuan	4.8

### Measurement scales

#### Community cohesion scale

Community cohesion was measured by the Community Cohesion Scale adapted from Sampson’s Collective Efficacy Scale (6). This scale consists of 4 items, such as, “People in my community generally get along well with each other”, “My community is a safe place.” Items are scored on a 5-point Likert scale from 1 (strongly disagree) to 5 (strongly agree), with higher scores indicating higher community cohesion. The Cronbach’s α for this study was 0.87.

#### The patient health questionnaire-9

Depression was assessed with the 9-item Patient Health Questionnaire Depression Scale (PHQ-9) (32) It is a 9-item self-report questionnaire in which participants were asked to rate how they felt in the previous 2 weeks. Each question is scored from 0 (not at all) to 3 (nearly every day) with a resulting range of 0 to 27. Higher scores indicate severity of depression, with a recommended cut-off of 10 or above for distinguishing between clinical and non-clinical populations. The Cronbach’s α for the questionnaire in this study was 0.91.

#### Social support questionnaire

The 4-item Social Support questionnaire was developed by the Chinese Academy of Social Sciences (CASS) to measure participants’ social support. Participants gave responses on a seven-point Likert scale from 1 (no support at all) to 7 (complete support). Higher average scores indicate a higher level of social support. The total Cronbach’s α for the questionnaire in this study was 0.92.

#### Connor and Davidson resilience scale

Psychological resilience was assessed using the Connor and Davidson Resilience Scale (CD-RISC) (20). The CD-RISC contains 25 items across 5 dimensions, all of which carry a 5-point range of responses from 1(not true at all) to 5(true nearly all of the time). The scale is rated based on how the subject has felt over the past month, with higher scores reflecting greater resilience. This scale has been widely applied and validated in Chinese clinical practice, with Cronbach’s α ranging from 0.81 to 0.91 (33, 34). It showed a Cronbach’s alpha of 0.81 in the current study. Questionnaire validity indicators show good fit (χ2/df = 3.53, CFI = 0.90, TLI = 0.89, SRMR = 0.04, RMSEA = 0.07).

### Data analysis

Mediation effects were tested using the bias-corrected percentile Bootstrap with 5000 repetitions of resampling in the study method (35). The mediation model analysis was performed using SPSS 25.0 in combination with the SPSS Macro PROCESS (36). Pearson correlation analysis was employed to explore relationships between variables. Further, a path analysis was conducted to assess a hypothesized mediation model concerning the effect of various factors on depression.

## Results

### Common method bias test

The Harman’s single-factor test was conducted to test the common method bias of the used items in the self-reported scales of community cohesion, social support, resilience, and depression. The results showed that the first common factor accounted for 34.70% of the total variance, which was below the critical threshold of 40% (37). Confirmatory factor analysis was used to further examine common method bias using Mplus8.3, and the fit result of the single-factor model was very poor (χ^2^/*df* = 12.90, CFI = 0.62, TLI = 0.60, SRMR = 0.11, RMSEA = 0.11). However, the indicators in four-factor model show good fit (χ^2^/*df* = 4.74, CFI = 0.88, TLI = 0.87, SRMR = 0.05, RMSEA = 0.06). The findings indicated that there was no significant common method bias in the current study.

### Correlation analysis

Our correlational analysis revealed significant relationships among community cohesion, social support, resilience, and depression. Specifically, community cohesion negatively correlated with depression scores, indicating that greater community cohesion was associated with less depression (*r* = -0.21, *p* < 0.01). Moreover, social support scores were negatively linked to depression scores (*r* = -0.27, *p* < 0.05), and positively correlated with psychological resilience (*r* = 0.32, *p* < 0.01), suggesting that social support contributes to less depression and high psychological resilience. Psychological resilience was negatively correlated with depression scores (*r* = -0.37, *p* 185 < 0.01), indicating that higher psychological resilience is associated with less depression. Also, community cohesion is significantly related to social support (*r* = 0.39, *p* < 0.001) and psychological resilience (*r* = 0.35, *p* < 0.001) (**Table 2**).

**Table 2:** Descriptive statistics and correlation analysis for all variables.

Variables	*M*	*SD*	1	2	3
1 Community Cohesion	4.16	0.78			
2 Social Support	5.23	1.07	0.39^***^		
3 Psychological Resilience	3.51	0.66	0.35^***^	0.32^**^	
4 Depression	1.84	0.58	-0.21^**^	-0.27^*^	-0.37^**^

^*^p<0.05, ^**^p<0.01, ^***^p<0.001; M, Mean; SD, Standard Deviation.

### Mediating role of social support and psychological resilience

**Figure 1:**
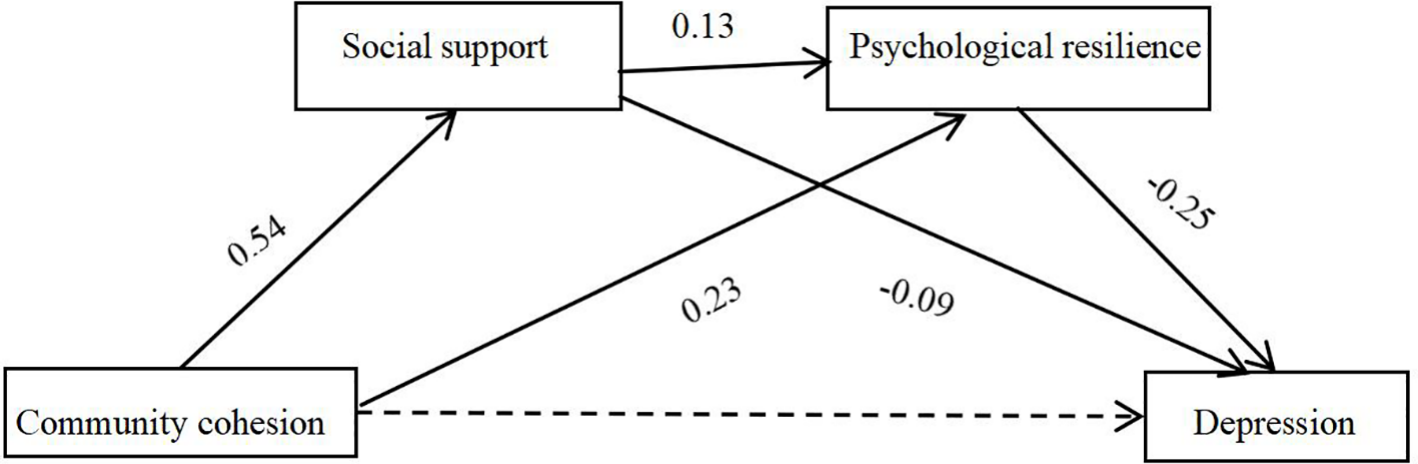
Mechanism model of the effect of community cohesion on depression.

Using the SPSS Macro PROCESS developed by Hayes (36), the mediating roles of social support and psychological resilience in the relationship between community cohesion and depression among adults was analyzed, while controlling for gender, age, education years and income Regression analysis results (as presented in **Table 3**, **Figure 1**) was shown that community cohesion positively affected social support (*β* = 0.54, *t* = 13.63, *p* < 0.001, 95% *CI* = [0.46, 0.62]); Social support (*β* = 0.13, *t* =6.91, *p* < 0.001, 95% *CI* = [0.09, 0.17])and community cohesion (*β* = 0.23, *t* = 8.79, *p* < 0.001, 95% CI = [0.18, 0.28]) had positive effect on psychological resilience. Both social support (*β* = -0.09, *t* = -5.05, *p* < 0.001, 95% *CI* = [-0.12, -0.05] )and psychological resilience (*β* = -0.25, *t* = -8.82, *p* < 0.001, 95% *CI* = [-0.30, -0.19]) negatively affected adult depression. Community cohesion demonstrated no significant direct effect on depression (*β* = -0.04, *t* = -1.67, *p* > 0.05, 95% CI = [-0.09, 0.01]), but a significant total negative effect on depression (*β* = -0.16, *t* = -7.17, *p* < 0.001, 95% *CI* = [-0.21, -012]).

**Table 3:** Regression analysis of variable relationship.

Regression variables		Goodness-of-fit index			Significance of coefficients		
Dependent variable	Independent variable	*R*	*R^2^*	*F*	β	*t*	*p*
Social Support	Gender	0.4	0.16	37.99	-0.14	-1.90	0.058
	Age				-0.004	-1.21	0.228
	Education years				-0.008	-0.52	0.600
	Income				0.007	0.09	0.928
	Community Cohesion				0.54	13.63	0.000
Psychological Resilience	Gender	0.43	0.19	38.82	-0.09	-2.09	0.037
	Age				0.003	1.31	0.191
	Education years				0.01	1.15	0.249
	Income				0.14	3.10	0.002
	Community Cohesion				0.23	8.79	0.000
	Social Support				0.13	6.91	0.000
Depression	Gender	0.43	0.18	31.95	0.03	0.70	0.484
	Age				-0.007	-4.00	0.000
	Education years				-0.01	-1.40	0.161
	Income				-0.04	-0.94	0.347
	Community Cohesion				-0.04	-1.67	0.095
	Social Support				-0.09	-5.05	0.000
	Psychological Resilience				-0.25	-8.82	0.000

Gender: Male=0, Female=1.

Social support and psychological resilience jointly mediated the relationship between community cohesion and depression, with a total mediation effect value of -0.12, accounting for 75% of the total effect (-0.12/-0.16). Specifically, the mediation effect comprised three indirect pathways: Pathway 1 (Community Cohesion → Social Support → Depression) demonstrated a significant mediating effect of -0.05 (95% *CI* [-0.07, -0.03]), accounting for 31.25% of the total effect (-0.05/-0.16); Pathway 2 (Community Cohesion → Psychological Resilience → Depression) showed a significant mediating effect of −0.06(95% *CI* [-0.08, -0.04]), accounting for 37.50% of the total effect (-0.06/-0.16); Pathway 3 (Community Cohesion → Social Support → Psychological Resilience → Depression) produced a significant mediating effect of −0.02(95% *CI* [-0.03, -0.01], accounting for 6.25% of the total effect (-0.02/-0.16) (**Table 4**).

**Table 4:** Mediation analysis of social Support and psychological resilience.

Mediated pathway	Mediator effect	*95% CI*	Effect size
Community cohesion→Social Support→Depression	-0.05	[-0.07, -0.03]	31.25%
Community cohesion→ Psychological Resilience →Depression	-0.06	[-0.08, -0.04]	37.50%
Community cohesion→ Social Support→ Psychological Resilience→ Depression	-0.02	[-0.03, -0.01]	6.25%

## Discussion

### Community cohesion and depression

Correlation analysis has shown that there was a negative relation between community cohesion and depression, which validated Hypothesis 1 of this study. Consistent with previous studies (7, 9), the finding suggested individuals living in higher community cohesion may have less risk of getting depression. According to Social Disorganization Theory, cohesion among members in a neighborhood, is a proximate mechanism through which neighborhood may affect individuals (6). On the one hand, communities with high cohesion provide residents with more resources and convenient facilities, and this can make residents adopt and reinforce healthy behaviors (46). On the other hand, highly cohesive communities can reduce individuals’ sense of self-reliance by offering more opportunities for interaction and enhancing the sense of purpose (21, 38). Moreover, a socially cohesive community can enhance individuals’ restorative experiences (39), thereby exerting a protective effect on their physical and mental health. Regression analysis revealed that the total effect of community cohesion on depression was significant, but the direct effect was not. This finding helps explain Abada et al.’s observation that perceived cohesion was unrelated to depression (40). We believe the reason is although community cohesion does not directly affect depression, it may exert an indirect influence through mediating variables such as social support and psychological resilience (as demonstrated in this study).

### The mediating effect of social support

Consistent with prior research (41), our findings revealed that social support mediated association between community cohesion and depression, which validated Hypothesis 2 of this study. According to the social capital theory (42), communities with strong cohesion are more likely to develop “bridging social capital,” which facilitates cross-group support. Social support can encourage positive health behaviors through established social norms (41) and enhance self-efficacy, or promote confidence (43), which are protective factors against individual depression.

### The mediating effect of psychological resilience

Our finding demonstrated that psychological resilience served as a significant mediating factor in the relationship between community cohesion and depression, which validated Hypothesis 3 of this study. This finding aligns with prior research (25, 26), which suggests that in a community with higher cohesion, individuals are more likely to develop greater psychological resilience, which is a protective factor against depression (44). This is in accordance with findings that children who lived in relatively high crime neighborhoods with low social cohesion were less likely to be resilient versus non-resilient to maltreatment (45). Ruiz and his colleagues confirmed that the association between low social cohesion and increased depressive symptoms operated via a psychological, but not a health behavioral, pathway (10). Our study extends their work by investigating how community cohesion influences depression through psychological resilience.

### Serial mediation of social support and psychological resilience

This study revealed a weaker but significant chain mediation of social support and psychological resilience, which validated Hypothesis 4 of this study. According to Conservation of Resources Theory (29), community cohesion may provide social support and a climate of encouragement, feelings of being understood and mutual respect, which in turn, help individuals to adopt healthy behaviors, enhance sense of control or mastery and use active coping strategies (14, 46, 47). The findings support Bronfenbrenner’s Ecological Systems Theory (48) and provide a comprehensive mechanism through which community cohesion fosters social support, and social support bolsters resilience, ultimately alleviating depression.

In summary, Individuals in communities with high cohesion can establish good social relationships with others based on mutual trust, gain more emotional and material social support among residents. When they encounter difficulties and stress in life, they will actively seek neighborhood support or develop the ability to recover quickly from difficulties, which can prevent or alleviate depression. While this model suggested in this study can reveal the mechanisms underlying the prevention or alleviation of depression well, it also has phenomena that it cannot explain. For instance, in collectivist cultures, individuals in communities with high cohesion are more sensitive to group norms and thus tend to suppress their own emotions, which will cause individuals to develop negative emotions (49) Therefore, this model will be improved in future research.

## Policy recommendation

The mediating model of community cohesion’s influence on depression through social support and psychological resilience offers critical insights into the occurrence and prevention of depression from the perspective of socio-environmental factors with individual psychological processes. This mechanism highlights that effective mental health promotion requires both the construction of a supportive social environment and the cultivation of individual psychological capacities for maximizing depression prevention and alleviation.

In the context of social transformation, rural-urban mobility, and digitalization, interpersonal bonds have progressively weakened. It is increasingly vital to strengthen the cultivation of community cohesion. By doing so, we can foster social interactions, amplify support networks, leverage collective resources during crises, and facilitate psychological resilience, ultimately mitigating distress from depression.

## Limitation

Firstly, the sample is from Chinese cities online, and most participants are highly educated, so regional homogeneity, voluntary participation and the online nature of the application limit the representativeness of the sample. Moreover, due to the special circumstances of the pandemic period, the generalization of the results is limited.

Secondly, since this study entirely adopts self-reported data and is collected at a single time point, there may be participants’ response bias due to social desirability concerns and common method bias. Besides, there may be potential bidirectional relationships between variables in this study. For instance, the individuals with higher depression levels may perceive lower community cohesion. Therefore, future research should employ experimental or longitudinal designs to further investigate the 305 association between community cohesion and depression.

Thirdly, this study examines social support derived from diverse individuals andorganizations withoutdistinguishingbetween support types (e.g., instrumental support). Future research should specifically investigate how distinct categories of social support as well as community cohesion and psychological resilience influence depression.

## Conclusion

(1) Community cohesion shows significant positive correlations with both socialsupport andpsychological resilience, while exhibiting a significant negative correlation with depression.Social supportand psychological resilience are also positively correlated, and both demonstrate significant negative associations with depression.
(2) Community cohesion does not directly affect depression but indirectly affects it through three mediation pathways: the mediating effect of social support; the mediating effect of psychological resilience; the serial mediation effect of social support and psychological resilience.

## Data Availability

The datasets presented in this article are not readily available because The data is currently still in the unpublished stage. Requests to access the datasets should be directed to WJ, wang_jx@cass.org.cn.
